# Arbitrary Use of Insulin and Its Consequence

**Published:** 2018-08

**Authors:** Masoumeh SHOHANI, Hamed TAVAN

**Affiliations:** 1. Dept. of Nursing, Faculty of Allied Medical Sciences, Ilam University of Medical Sciences, Ilam, Iran; 2. Dept. of Nursing, Students Research Committee, Ilam University of Medical Sciences, Ilam, Iran

## Dear Editor-in-Chief

Diabetes is one of the most common diseases in the country and has negative short and long-term effects on body systems (brain, heart, and kidneys) (1–3). The prevalence of diabetes is high in Iran and 1 out of 20 Iranians afflicted with diabetes, but the problem is most people are not aware that they have diabetes leading to irreparable damages (4–6).

A 62-yr-old patient with a diagnosis of diabetes type 2 was admitted to Shahid Mostafa Khomeini Hospital, in Ilam, western Iran in 2016 and knew that he had diabetes, but arbitrary consumed insulin in two ways. He firstly changed the types of used insulin, regular and NPH insulin into lantos and nova mix and secondly changed dose of insulin which had consequences on the patient. He firstly encountered loss of vision and did not go to hospital, when he went to the hospital he was almost blind. Arbitrary use of insulin not only led to blindness but also he severely infected with uncontrolled high blood sugar after a year resulting to foot amputation patients and after 6 months the patient died.

Diabetes is a serious disease and ignoring it causes serious complications (kidney damage, amputation, and blindness) (7). Diabetes is a disease that can be prevented from its progress if blood sugar precisely controlled and if arbitrary used leads to irreparable effects on the infected person. Suspected patients should identify and screening tests should perform for first degree relatives and close relatives of patients due to the hereditary nature of this disease, after the diagnosis of diabetes, the diabetes patients and families should be informed with all complications of diabetes and symptoms in cases of hypoglycemia and hyperglycemia to perform proper action. Moreover, proper training should be taken to familiarize patient and its family on how to inject insulin and its places, how to regulate its dosage during day and overnight and to monitor blood sugar. In the case of new symptoms of the disease, the patient should refer to endocrinologist and do not arbitrarily change the insulin. The patient diet should properly adherer to health care provider directions and exercise consumes sugar in the body and can improve disease.

Fully compliance with the above-mentioned instructions, the patient can prevent progress of diabetes and any arbitrary actions may lead to unpleasant event and even death.
